# Short Videos Turn Everyone into Bearers of Traditional Sports and Games: A Mixed-Methods Study from China

**DOI:** 10.3390/bs15050637

**Published:** 2025-05-07

**Authors:** Shuangshuang Liu, Yifan Zuo, Sirong Chen, Rob Law, Jiabao Cui

**Affiliations:** 1 School of Sports, Shenzhen University, Shenzhen 518060, China; 2300371016@email.szu.edu.cn (S.L.); yifanzuo@szu.edu.cn (Y.Z.); 2 Asia-Pacific Academy of Economics and Management, University of Macau, Macao SAR, China; sirong.chen@connect.um.edu.mo (S.C.); roblaw@um.edu.mo (R.L.); 3 Department of Integrated Resort and Tourism Management, Faculty of Business Administration, University of Macau, Macao SAR, China

**Keywords:** traditional sports and games, responsible behavior, value-belief-norm theory, theory of planned behavior, short video app users, partial least squares structural equation modeling, fuzzy-set qualitative comparative analysis

## Abstract

The emergence of short video applications (“apps”) has facilitated the dissemination, inheritance, and protection of traditional sports and games (TSGs). However, the effectiveness of these apps in raising public awareness and responsibility toward the preservation and heritage of TSGs has received insufficient research attention. This study constructs a theoretical model based on value-belief-norm theory and the theory of planned behavior, employing both PLS-SEM and fsQCA methods to empirically analyze 417 questionnaires. The results indicate that the PLS-SEM method identifies key factors influencing users’ responsible behaviors toward TSGs on short video apps, along with the complex and interdependent relationships among these factors. The fsQCA method reveals the intricate interactions and nonlinear effects of the antecedents on users’ responsible behaviors and identifies six configurations that drive high-level TSG responsible behaviors among users. This paper extends research on public responsible behaviors concerning TSGs and provides important practical guidance for government managers, inheritors, and conservation entities in the protection and heritage of TSGs.

## 1. Introduction

As essential components of intangible cultural heritage, traditional sports and games (TSGs) are currently facing a severe survival crisis because many of them have now faded into obscurity. Meanwhile, others teeter on the brink of extinction, signifying an urgent need to implement intervention and rescue efforts from various sectors of society. In China, a country rich in cultural heritage, as many as 1356 representative items of TSGs exist (Zuo et al., 2023); therefore, their protection and preservation are tasks that can be quite daunting. However, not all these items actually receive the timely and effective attention and protection that they need. In response to this challenge, the Chinese government is actively promoting the use of new media technologies to revitalize TSGs, emphasizing the need to strengthen their dissemination and popularization. This undertaking includes diversifying communication methods, expanding dissemination channels, and encouraging various media platforms to participate comprehensively in the promotion of TSG content. In this context, short video platforms have played a significant role in disseminating traditional culture (Ning, 2025) and have shown considerable potential in enhancing public awareness and recognition of TSGs.

Short videos, with their characteristic freedom of creation, have been combined with the performative value of TSG, gradually becoming one of the important channels for TSG dissemination. Short videos have been shown to increase the exposure of TSGs, enhance users’ awareness of the current state of TSG protection, educate them to understand their responsibilities, and ultimately stimulate protective actions (X. Chen & Xian, 2022). Short videos also provide new possibilities for cultivating the public’s responsible behaviors toward intangible cultural heritage.

Furthermore, the academic community has consistently focused on how to enhance the public’s awareness and sense of responsibility for protection and inheritance, especially in the field of TSG, where extensive discussions have taken place (Hsu et al., 2022). However, to propose suggestions and strategies for enhancing public TSG responsible behaviors, with particular emphasis on exploring the communicative power of short video platforms, existing research and practice tend to focus on theoretical discussions, often starting from practical difficulties (Du & Luo, 2022). To date, research on using short videos to enhance the public’s awareness and sense of responsibility for TSG protection and inheritance is mostly superficial, with the deeper logical mechanisms somewhat neglected.

In previous studies, value-belief-norm theory (VBN) and the theory of planned behavior (TPB) have often been employed to explain and predict prosocial behaviors, particularly within the domain of ecological civilization (Le et al., 2021; Wang et al., 2018). Given that behaviors related to TSG responsibility are a specific form of prosocial behaviors and that the preservation and transmission of TSGs fall under the scope of ecological civilization, the integration of VBN and TPB offers a new perspective for constructing an explanatory framework for TSG responsible behavior. Based on this context, the present study integrates VBN and TPB to empirically explore the mechanisms underlying the formation of TSG responsible behaviors among short video app users.

First, this study employs partial least squares structural equation modeling (PLS-SEM) to verify the influence effects of antecedent variables on TSG responsible behaviors among short video app users. On this basis, it further adopts fuzzy-set qualitative comparative analysis (fsQCA) to explore the antecedent variables’ configurational effects on TSG responsible behaviors among short video app users. This study also identifies the necessary and sufficient conditions, thereby providing a reference for stimulating TSG responsible behaviors among short video app users.

## 2. Literature Review and Research Hypotheses

### 2.1. Traditional Sports and Games

**Figure 1:**
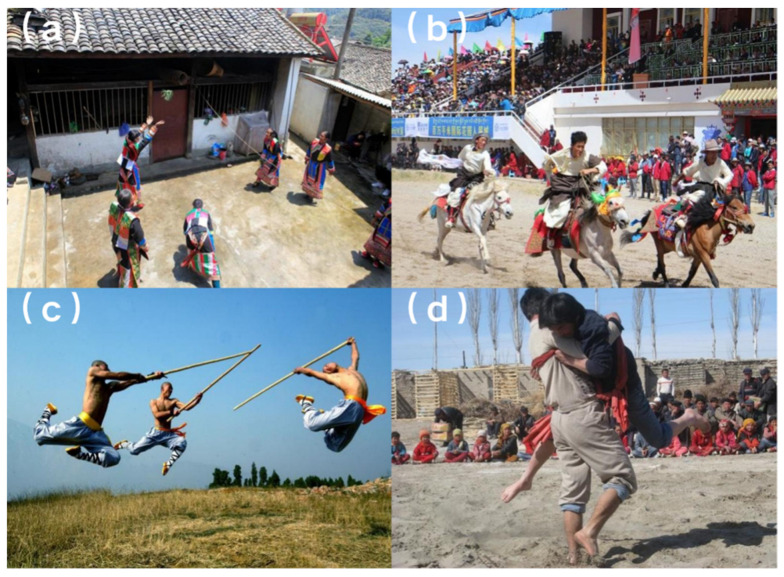
(**a**) Yunnan Lisu Dudada: a net-separation game somewhat similar to volleyball. (**b**) Tibet Qiangtang Qiaqing Horse Racing Festival: the largest sports competition event in the northern Tibetan region. (**c**) Henan Shaolin Kung Fu Chongtian Staff Formation: a staff attack technique used by warrior monks in actual combat. (**d**) Xinjiang Qelixi: a traditional Uyghur wrestling competition with no age or weight divisions. These photos were obtained from field research.

TSGs are sports activities practiced worldwide for centuries before the advent of modern sports. Unlike modern sports, TSGs were often confined to specific geographical areas (Renson et al., 1997). Figure 1 illustrates four common TSGs in China. As an essential part of human cultural heritage and intangible sports cultural heritage, they carry rich historical and cultural values (Tautges et al., 2011; Theeboom & De Knop, 1997). The importance of TSGs to China’s cultural heritage and the necessity of their protection are recognized by the United Nations Educational, Scientific, and Cultural Organization (UNESCO; Zuo et al., 2023). However, with rapid changes in lifestyles and production methods, the deterioration of TSGs has accelerated due to their inherent intangibility, fragility, and the characteristics of its transmission, with many items either disappearing or on the brink of extreme endangerment (Riordan, 1986; Zuo et al., 2023). In response to this challenge, a global exploration of digital technologies is established in the protection of ethnic TSG, including innovative inventory compilation methods using online platforms such as “eNanda Online” (Marschall, 2014).

### 2.2. TSG Responsible Behavior

The term “TSG responsible behavior” originates from “heritage responsible behavior”, a compound concept that integrates heritage responsibility with responsible behaviors. This concept refers to the responsibilities individuals or institutions bear during the cognition, interpretation, and reproduction stages of heritage values (Ju & Cheng, 2023). Currently, research on responsible behavior has focused on environmental protection and corporate marketing, involving diverse aspects, such as environmental, economic, ethical, and community responsibilities. Scholars have recognized that adopting heritage protection measures in production and living directly affects the sustainable development of heritage. Based on this understanding, the concept of heritage responsible behavior was proposed, defined as the moral responsibility residents bear for the protection of heritage resources within their living areas (Ju & Cheng, 2023). Heritage responsible behaviors generally fall into the following two main categories: (1) material cultural heritage responsible behaviors, which focus on the protection of tangible cultural heritage, such as preserving historical sites, and (2) intangible cultural heritage responsible behaviors, which are relatively weaker in terms of publicity and public awareness. The present study’s focus on TSG responsible behavior falls under the second category.

TSG responsible behaviors entail a series of actions and attitudes aimed at protecting, promoting, and inheriting TSGs, including (1) knowledge acquisition and deep understanding, involving deep learning of the history, cultural significance, and practices of TSGs, as well as actively exploring and understanding their cultural and historical values; (2) social interaction and dissemination, involving discussing and sharing information on TSGs within social circles and through platforms, such as social media, to expand their influence and awareness; (3) active participation and support, involving individual participation and support for TSG protection activities, including attending public events, practices, training, or volunteering; and (4) sense of responsibility and awareness of protection, including recognizing the importance of protecting TSGs and taking actions to maintain and inherit these cultural treasures, serving as a core driver for individual active participation (Gursoy et al., 2019).

### 2.3. Cultural Worldview

Previous research has suggested that attitudes determine the occurrence of behaviors, reflecting individuals’ evaluations of others, things, and ideas (Ajen & Fishbein, 2005). A cultural worldview (CW) is a kind of attitude toward culture, representing the fundamental viewpoints and attitudes people hold, including beliefs, concepts, and perceptions; such a definition reflects the common cognition and ideas formed in cultural heritage and social development (Choi et al., 2009). A country’s CW is a product that is continually accumulated by a nation over its history. Even within the same cultural background, differences in individuals’ attitudes toward culture persist, further influencing their cognitive processing, attitude preferences, and specific behavioral strategies regarding culture (Piff et al., 2012).

### 2.4. Value-Belief-Norm Theory and Heritage Responsible Behavior

VBN theory is a psychological construct that specifically applies Fulton’s hierarchical model (Choi et al., 2009). VBN theory links value orientation, belief cognition, consequence awareness, responsibility attribution, and behavioral norms through a causal chain. This theory, often used to analyze prosocial behaviors, hypothesizes that an individual’s prosocial behavioral intentions and actions are determined by personal norms, which are influenced by responsibility attribution.

In the VBN model for short video app users’ TSG responsible behaviors, “values” refer to the cultural values of such users, reflecting their attitudes toward activities and issues related to TSGs after watching related short videos. Andy and others (as cited in Choi et al., 2009) have proposed a CW scale comprising the following four factors: cultural linkage, recognition of cultural value, loss sensitivity, and reservation consistency, to measure people’s cultural attitudes. “Beliefs” include two variables: (1) awareness of consequence, referring to an individual’s awareness of the potential consequences of actions that could benefit or harm society and an individual’s prediction and evaluation of the possible results of specific actions or decisions; and (2) ascription of responsibility, involving attributing negative outcomes to oneself, feeling responsible for these consequences, and believing that human actions can prevent or mitigate potential negative outcomes. Meanwhile, “norms” refer to moral norms or the moral obligations requiring specific behaviors.

Based on this theory, TSG protection and inheritance awareness is a cognitive activity concerning TSG protection, mainly measuring people’s attention to, acceptance of, and personal efforts toward TSG protection and inheritance. This study hypothesizes that short video app users can enhance their awareness of TSG protection and inheritance by watching related short videos. Moreover, those with such awareness are likely to consider protecting TSGs as a prosocial behavior, believe that protecting TSGs helps promote and inherit excellent Chinese traditional culture, foster sustainable social development, and consider the protection and inheritance of TSGs as their responsibility. In this context, users who have watched TSG short videos are likely to form cognitive outcomes and ascribe responsibility for TSGs, thereby establishing specific moral norms. Therefore, the following hypotheses are proposed:

**H1a.**  *The TSG relevance of short video app users positively affects their awareness of consequences.*

**H1b.**  *The TSG recognition of cultural value by short video app users positively influences their awareness of consequences.*

**H1c.**  *The loss sensitivity to TSGs by short video app users positively affects their awareness of consequences.*

**H1d.**  *The preservation consistency toward TSGs by short video app users positively affects their awareness of consequences.*

**H2.**  *Users’ TSG awareness of consequences positively influences their ascription of responsibility for TSG issues.*

In accordance with VBN theory, beliefs directly influence norms. In the present study, beliefs manifest as the ascription of responsibility for TSG issues by short video app users. Past research has found that ascription of responsibility can directly influence personal norms or moral norms and has an enhancing effect. Based on this assumption, the following hypothesis is proposed:

**H3.**  *Users’ ascription of responsibility for TSG issues positively influences their TSG-related moral norms.*

Individual behavior is generally influenced by personal norms, particularly moral norms, which set standards for individuals’ behavioral decisions (Ding et al., 2018). Generally, short video app users who have watched TSG short videos feel greater moral constraints than others, and moral norms enhance their willingness to engage in responsible behaviors for protecting and inheriting TSGs. Based on this finding, the following hypothesis is proposed:

**H4.**  *Users’ TSG moral norms positively influence their TSG responsible behaviors.*

### 2.5. Theory of Planned Behavior and Heritage Responsible Behavior

TPB is also a concrete application of psychological constructs from Fulton’s hierarchical model, linking the concepts of perceived behavioral control, subjective norm, attitudes, behavioral intentions, and behaviors through a causal chain (Ajzen, 1991). TPB is frequently used to describe how individuals change their behaviors and serves as a theoretical basis to facilitate hypothesis model construction.

The core principle of TPB suggests that attitudes toward behaviors, subjective norms, and perceived behavioral control can effectively influence the formation of behavioral intentions, which, in turn, trigger actual behaviors (Ajzen, 1991). Previous studies on responsible behavior, based on TPB, often yield good explanatory or predictive outcomes (T.-I. Han & Stoel, 2017), demonstrating TPB’s potential in forecasting TSG responsible behaviors.

If users of short video apps hold positive attitudes toward TSG responsible behaviors, feel strong social expectations, and believe they have the capability to achieve TSG protection, the likelihood of exhibiting TSG responsible behaviors increases. Past research has also adequately confirmed the associations among TPB variables, indicating that these variables play a crucial role in predicting individual decision-making processes (Ajzen, 1991). Therefore, the following hypotheses are proposed:

**H5.**  *TSG subjective norms positively influence TSG responsible behaviors.*

**H6.**  *Users’ attitudes toward TSG responsible behaviors positively influence their TSG responsible behaviors.*

**H7.**  *Perceived control over TSG responsible behaviors positively influences TSG responsible behaviors.*

### 2.6. Integrating VBN and TPB Models

Both VBN and TPB are specific applications of psychological constructs within Fulton’s hierarchical model, originating from the same framework but adopting different perspectives to incorporate individual motivations into their behavior decision-making processes. VBN is based on values and links beliefs and moral norms; whereas the TPB model constructs the need for specific attitudes, subjective norms, and perceived behavioral control when individuals perform specific actions (Ajzen, 1991). Owing to their complementary characteristics, VBN and TPB are often integrated and combined in previous studies. The integrated model considers variables from both VBN and TPB, thus avoiding the shortcomings of single-theory models and providing a comprehensive explanation of users’ attitudes and motivations toward TSG responsible behaviors. This integrated model not only offers a comprehensive perspective from intrinsic values to external social norms but also holds essential guidance for devising effective TSG protection strategies. By thoroughly understanding individual motivations and behavioral intentions, more effective interventions to foster a sense of responsibility and proactive behaviors can be designed. Existing research indicates that awareness of consequence plays a key role in influencing pro-social attitudes toward behavior and perceived behavioral control (Bamberg & Möser, 2007), while subjective norms play a crucial role in the formation of moral norms and responsible behaviors (Onwezen et al., 2013). Based on these insights, the following hypotheses are proposed:

**H8.**  *TSG awareness of consequence positively influences perceived control over TSG responsible behavior.*

**H9.**  *TSG awareness of consequence positively influences attitudes toward TSG responsible behavior.*

**H10.**  *TSG subjective norms positively influence TSG moral norms.*

**Figure 2:**
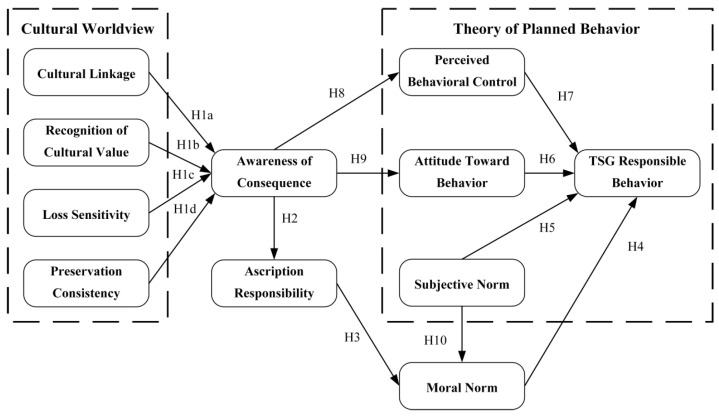
Hypothesis model.

The hypothetical model of TSG responsible behavior among short video app users is illustrated in Figure 2.

## 3. Research Design

The data analysis of this study is divided into two parts. First, the proposed theoretical model is validated using the PLS-SEM approach to examine the relationships within it, followed by an exploration of the complex causal relationships between variables within the theoretical model using the fsQCA method.

PLS-SEM is a method based on partial least squares and structural equation modeling and is designed for processing and interpreting multivariate data. It combines the advantages of both PLS and SEM, enabling dimension reduction and prediction, as well as testing causal relationships between variables. This method is particularly robust when testing complex models and indirect effects (Barnes et al., 2020). Therefore, it is suitable for validating the complex theoretical model proposed in the present study, providing significant practical value in understanding how different factors influence responsible behaviors and the potential outcomes resulting from these factors. This study also uses Smart PLS 4.0 software for analysis. In comparison, fsQCA allows for the exploration of multiple causal pathways rather than assuming a single causal path. When studying responsible behaviors toward TSGs, the factors influencing user behavior are often diverse, and fsQCA can reveal how different combinations of variables interact to produce specific behavioral outcomes. This method helps us understand how multiple factors interact in real-world contexts, which may not be fully captured by PLS-SEM (Fiss, 2007).

These two methods complement each other. While PLS-SEM provides quantitative path estimates, fsQCA helps reveal the multifaceted combinatory effects across different contexts. By combining them, the present study is able to comprehensively explore the causal mechanisms influencing responsible behaviors toward TSGs, thus offering richer theoretical and practical insights into the topic.

### 3.1. Study 1

#### 3.1.1. Variable Measurement

The questionnaire used a seven-point Likert scale, ranging from “strongly disagree” (=1) to “strongly agree” (=7). To ensure the content validity of the scale, this study followed a standardized procedure for questionnaire development based on prior research. The item validity of each variable’s scale was assessed by two scholars, two graduate students, and two undergraduates, with all measurement items derived from existing research. Appendix A presents all the measurement items. The participants were required to evaluate the content and understandability of the measurement items and suggest edits and improvements to enhance the clarity, readability, and content validity of the items. The expert group also determined whether redundancy existed among items and proposed improvements to construct quota items. In this case, the aims were to minimize semantic differences in scales from international studies and ensure that the items were more aligned with the practical realities of the research.

#### 3.1.2. Data Collection

The questionnaire consisted of the following three parts: an introductory statement, the main body, and demographic information. The introduction describes the purpose of the survey, informed consent, and other details. The main body is divided into questions measuring variables related to video materials and theoretical models, featuring a 30 s TSG Douyin video “https://v.douyin.com/ieXd3Bm5/?utm_campaign=client_share&app=aweme&utm_medium=ios&tt_from=copy&utm_source=copy (accessed on 20 February 2024)”, accompanied by lie detection questions and a video summary. The demographic section collects data on the participants’ gender, age, and educational background. After the initial questionnaire design, two pilot tests were conducted to further validate the questionnaire. Based on feedback and anticipated survey outcomes, adjustments and optimizations were made, thus finalizing the questionnaire content. The study utilized convenience sampling and distributed the questionnaire online via Credamo “https://www.credamo.com/#/” (accessed on 29 April 2025). The questionnaire link was shared in various WeChat groups, and a red envelope was added at the end of the survey to incentivize possible responses. Out of a total of 452 distributed questionnaires, 417 valid questionnaires were obtained after screening out invalid responses through lie detection questions and survey completion time, ultimately yielding a validity rate of 92.25%. Among the respondents, 69.8% were male. Age distribution was as follows: 46.8% were aged 19–29 years, 30.5% were 30–39 years, 15.3% were 40–49 years, 4.8% were 50–59 years, and 2.6% were 60 years and above. Educational levels were as follows: 11.8% had a high school diploma, 62.6% held an associate/bachelor’s degree, and 25.7% had a postgraduate degree or higher.

#### 3.1.3. Study 1: Empirical Results and Analysis

1. Common Method Variance Test

This study employed descriptive statistical methods to analyze the characteristics of respondents’ answers, with metrics including the mean, standard deviation, skewness, and kurtosis (Wijaya et al., 2022). Table 1 provides a detailed summary of these statistical results. As shown in the table, the overall mean score across all measurement items was 5.182. Among these, PBC1 achieved the highest score (5.5), whereas CL3 obtained the lowest score (4.73), suggesting that respondents’ feedback was generally positive. Within each construct, no significant differences were observed in the items’ mean scores, and the distributions of standard deviations were similarly consistent. Moreover, both skewness and kurtosis were within an absolute value of 2 (Collier, 2020), thus supporting the assumption of a normal distribution.

To determine whether common method bias existed, Harman’s single-factor test was applied. Following the procedure, the first principal factor explained 39.979% of the variance, less than the critical standard of 50% (Podsakoff et al., 2003). Thus, no significant common method bias was found in this study.

#### 3.1.4. Reliability and Validity Tests

Reliability analysis was conducted using Cronbach’s alpha and composite reliability (CR), where CR measured the consistency of effects among various variables. As shown in Table 1, the Cronbach’s alpha coefficients for the latent variables ranged from 0.826 to 0.911, and the CR values ranged from 0.896 to 0.937, all exceeding the standard of 0.700, indicating the high reliability of the latent variables (Bagozzi & Yi, 1988). In terms of validity, the study assessed convergent validity by analyzing the factor loadings of each measurement item and the average variance extracted (AVE) of the latent variables. Data from Table 1 indicate that the factor loadings ranged from 0.812 to 0.921, all above 0.500; furthermore, the AVE values ranged from 0.724 to 0.831, also exceeding 0.500, thereby confirming good convergent validity of the measurement items (Bagozzi & Yi, 1988).

Discriminant validity was assessed using the Fornell–Larcker criterion, as illustrated in Table 2. This criterion tests discriminant validity by comparing the square root of the AVE with the correlation coefficients among latent variables. If the square root of the AVE is greater than the correlation coefficient among the variables (i.e., the diagonal values are greater than the off-diagonal values), it indicates good discriminant validity among the latent variables in the measurement model (Bagozzi & Yi, 1988). As shown in Table 2, the variables demonstrate discriminant validity.

2. Structural Model Analysis

**Figure 3:**
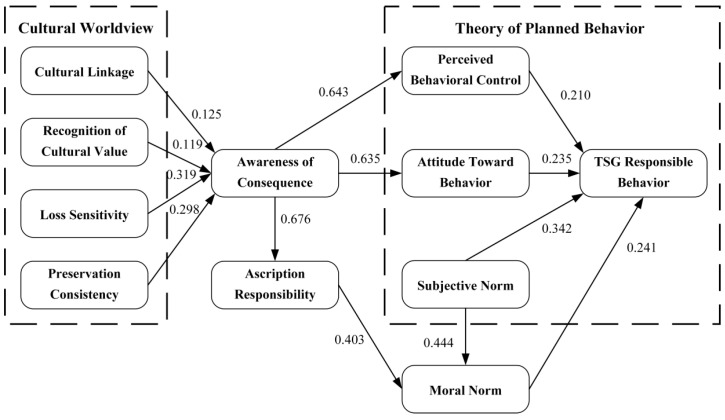
Hypothesis test results.

In the structural model evaluation of PLS analysis, the focus was primarily on estimating path coefficients and assessing R-squared values. Path coefficients reveal the intensity and direction of influence between latent variables, while R-squared values measure the extent to which exogenous latent variables explain endogenous latent variables, also reflecting the explanatory power of the model. In this study, we verified the effectiveness of the theoretical model and hypotheses by employing the visual Smart PLS 4.0 for PLS analysis, using a bootstrapping method with 5000 samples to determine the significance of path coefficients. The results are shown in Figure 3.

This study employed *Q*^2^ (predictive relevance), *R*^2^ (coefficient of determination), and goodness-of-fit (GOF) as key indicators for evaluating the structural model. As indicated in Table 3, the *R*^2^ values ranged from 0.404 to 0.617, suggesting that the model possesses moderate explanatory power. Furthermore, the *Q*^2^ values were between 0.321 and 0.482, all greater than 0, indicating the predictive relevance of the model. A GOF of 0.612, exceeding 0.36, demonstrates the model’s strong estimation capability.

As shown in Figure 3 and Table 4, the 13 hypotheses proposed in this study all passed the significance tests, indicating that the path relationships between variables can adequately explain the formation of TSG responsible behaviors among short video app users. Specifically, factors such as cultural linkage (*β* = 0.125, *p* < 0.05), loss sensitivity (*β* = 0.319, *p* < 0.05), preservation consistency (*β* = 0.298, *p* < 0.05), and recognition of cultural value (*β* = 0.119, *p* < 0.05) significantly influenced awareness of consequence. Moreover, moral norm (*β* = 0.241, *p* < 0.05), perceived behavioral control (*β* = 0.210, *p* < 0.05), attitude toward behavior (*β* = 0.235, *p* < 0.05), and subjective norm (*β* = 0.342, *p* < 0.05) all had significant effects on TSG responsible behaviors. Notably, awareness of consequence had strong direct impacts on ascription of responsibility (*β* = 0.676, *p* < 0.05), attitude toward behavior (*β* = 0.635, *p* < 0.05), and perceived behavioral control (*β* = 0.643, *p* < 0.05), thus indicating high predictive power to generate TSG responsible behaviors among short video app users.

3. Impact Effect Analysis

By calculating the direct and indirect effects, the total effects of each antecedent variable were determined, as shown in Table 5. The most significant effect on TSG responsible behaviors came from subjective norms, reaching 0.449, followed by awareness of consequence with an effect of 0.350. In comparison, ascription of responsibility had a smaller effect at just 0.097.

### 3.2. Study 2

Study 2 continued to use the same set of data from Study 1, although fsQCA (version 4.1) was used to further examine the data. As a qualitative analysis method based on set theory, fsQCA is capable of identifying causal relationships between various combinations of conditions and specific outcomes. Its principal operational steps include data calibration and the construction of a truth table.

First, prior to applying fsQCA, the raw measurements were calibrated into fuzzy-set membership scores ranging from 0 to 1, thereby quantifying the degree of case membership in a given set. Given that the original questionnaire used a seven -point Likert scale, and based on recommendations from existing literature, the present study applied the direct calibration method by establishing three anchor points for each condition variable in which a score of 7 indicated full membership, 4 represented the crossover point, and 1 denoted full nonmembership (Ling et al., 2021). To avoid the theoretical complications associated with cases exhibiting a membership score of exactly 0.5, a constant of 0.001 was added to all such cases (Greckhamer, 2016), thereby ensuring that the calibrated data more clearly reflected membership statuses. Furthermore, to maintain a good balance between the number of conditions and the number of cases (Jordan et al., 2011) and to avoid overfitting in QCA due to an excessive number of conditions, the present study incorporated cultural linkage, recognition of cultural value, loss sensitivity, and preservation consistency into the CW analysis.

Next, the construction of the truth table represented a critical step in fsQCA. The truth table enumerated all possible combinations of conditions and their associated outcomes, and representative configurations were identified by setting thresholds for the minimum acceptable frequency and consistency. Based on prior research findings and the specific context of this study, the minimum acceptable frequency was set at 2, suggesting that only configurations appearing in at least two cases within the sample were deemed representative. This step helped retain 94% of the observations and satisfied the criterion of preserving over 80% of the sample. In addition, a consistency threshold of 0.8 was adopted to ensure that the condition combinations had sufficient explanatory power regarding the outcome (Taheri et al., 2020).

In fsQCA analysis, the truth table typically yields three types of solutions: complex, parsimonious, and intermediate solutions. Complex solutions incorporate nearly all possible condition combinations and often result in lengthy and difficult-to-interpret expressions. Conversely, in the process of simplifying using logical remainders, parsimonious solutions disregard theoretical and empirical rationality, which might lead to the introduction of untenable simplifying assumptions. The intermediate solution, which serves as a middle ground between these two, simplifies the complex solution by retaining core conditions while avoiding the unwarranted assumptions inherent in the parsimonious solution. For the reasons stated above, the present study preferentially reported and interpreted the intermediate solution.

#### Study 2: fsQCA Results

The fsQCA necessary condition analysis (Table 6) revealed that the consistencies of the seven causal conditions all exceeded 90.9%, with coverage ranging from 84.11% to 88.39%. This finding indicates that these variables largely explain TSG responsible behaviors, thereby supporting the PLS-SEM analysis. Unlike the sufficient condition analysis in SEM path analysis, the necessary condition analysis in fsQCA identifies the prerequisite conditions for the formation of TSG responsible behaviors.

When the consistency reached above 90% and coverage exceeded 80%, it can be initially determined that the variable was a necessary condition for the outcome. However, based on further analysis, the X-Y scatter plots of the condition variables and the outcome variable revealed that most case points were distributed above the diagonal, indicating that none of the seven variables passed the necessary condition test (Vis & Dul, 2018). This result reveals the complexity of TSG responsible behaviors among short video app users. In particular, this finding suggests that such behaviors are not necessarily triggered by a single causal variable but require the combined effect of multiple variables.

As shown in Table 7, no single sufficient condition can trigger TSG responsible behaviors among short video app users. Nonetheless, six combinations of sufficient and consistent antecedent conditions have been identified to effectively predict TSG responsible behaviors among short video app users. Based on the existing research requirements for consistency thresholds (>0.75) (Greckhamer, 2016), these six antecedent configurations all meet the consistency standard, thus becoming sufficient conditions for the formation of TSG responsible behavior. An in-depth analysis of coverage revealed that the case coverage rate of these six antecedent configurations ranged from 77.15% to 79.33%. Overall, they collectively covered 88.22% of the result cases, indicating that they have strong explanatory power in understanding TSG responsible behaviors. In general, these configuration models jointly indicate the existence of an asymmetric and complex interaction between the antecedent variables of TSG responsible behaviors among short video app users. Under different configurations, the influence of certain specific antecedent variables may vary. For example, CW has a positive influence in Configurations 1, 2, 3, 5, and 6 but is absent in Configuration 4. Similarly, variables such as subjective norm, moral norm, attitude toward behavior, ascription of responsibility, perceived behavioral control, and awareness of consequence also display similar patterns. This finding indicates that the influence of antecedent variables on TSG responsible behaviors among short video app users is inconsistent or asymmetrical, with the effects of other variables in the model demonstrating complex interactions and synergistic effects.

This study primarily employed the methods of adjusting calibration thresholds and altering consistency thresholds to conduct robustness tests. First, in adjusting calibration thresholds, the values of 6, 4, and 2 were set as the thresholds for full membership, the crossover point, and full nonmembership, respectively (Pappas et al., 2016). After recalibrating the thresholds and repeating the original steps, the results revealed that the configurations remained completely consistent (Table 8). Furthermore, the consistency of the solution decreased by 0.0171 compared to before the adjustment, and the coverage of the solution decreased by 0.0056, indicating minimal changes.

In terms of altering consistency thresholds, the original consistency threshold was adjusted from 0.80 to 0.85 and 0.90, and the original steps were repeated for empirical analysis. The results revealed that the configurations remained completely consistent, and no changes occurred in the consistency and coverage of the solution compared to before the adjustment. Based on these findings, the results of this study can be considered robust.

## 4. Conclusion and Discussion

### 4.1. Theoretical Contributions

First, this study explored the impact effects and functional logic of TSG responsible behaviors among short video app users. Through theoretical deduction, empirical investigation, and testing, a fundamental theoretical framework for TSG responsible behaviors among short video app users was established. Thus, the paper expanded the research on intangible cultural heritage responsible behaviors and provided a new perspective on this phenomenon.

By employing an integrated VBN and TPB model, this study conducted an empirical investigation into the influencing mechanisms of TSG responsible behaviors among short video app users, further confirming the effectiveness of this integrated model in explaining prosocial behaviors (Al Mamun et al., 2024). The integration of the VBN and TPB models represents a fusion of cultural values with behavioral intentions, as well as social influence with personal responsibility. Specifically, VBN provides the backdrop of individual values and cultural identity, while TPB reveals how individuals transform these cultural values into concrete protective behaviors. As a medium for cultural dissemination and responsibility activation (Zhang, 2024), short videos enable audiences not only to understand the cultural values of TSG, but also effectively stimulate their sense of responsibility for protecting traditional culture (L. Chen, 2023), thus motivating them to engage in corresponding protective behaviors. On the one hand, the concepts of “cultural value identity” and “responsibility attribution” in VBN explain why individuals develop a sense of responsibility to protect TSG after watching short videos. On the other hand, “subjective norms” in TPB further elucidate that when audiences perceive support and expectations from society or groups, they are more likely to engage in protective behaviors. Especially on short video platforms, social norms are often expressed through interactive behaviors, such as comments, likes, or shares, thereby reinforcing individual responsibility and behavioral intentions.

Second, this study provides a multifaceted theoretical understanding of the formation of TSG responsible behaviors among short video app users. First, it identifies four key influencing factors for TSG responsible behavior: moral norms, perceived behavioral control, attitude toward behavior, and subjective norms. Then, it recognizes the complex mediation mechanisms through which individuals’ attitudes toward culture profoundly affect their cognition of the potential consequences of an activity, their evaluation of specific behaviors, their judgment of self-capability in completing certain tasks, and their personal moral values, which, in turn, promote the emergence of TSG responsible behaviors.

Furthermore, this study clarifies six equivalent combinations of antecedent variables that contribute to the generation of TSG responsible behaviors among short video app users. Additionally, it reveals the complex interactions and asymmetrical effects of the antecedent conditions on TSG responsible behavior, meaning that the influence of antecedent variables on TSG responsible behaviors is neither independent nor equivalent. The emergence of TSG responsible behaviors involves the interaction of multiple antecedent conditions, demonstrating the complexity of causal relationships. This finding corresponds with current research on enhancing the protection and inheritance of TSG through new media technologies (Gursoy et al., 2019) while providing empirical support for it.

Third, in deriving the universality of the integrated VBN and TPB model in the formation mechanism of TSG responsible behaviors, this study adopts a mixed research method, combining PLS-SEM with fsQCA to address the limitations of a single symmetric analysis (Fiss, 2007). In particular, Study 1 tested the validity of the hypothesized model using PLS-SEM, aiming to clarify the impact effects and functional logic of different antecedent variables on TSG responsible behaviors as the outcome variable. Study 2 employed fsQCA to examine the configurational effects of antecedent variables, deepening the understanding of the antecedent occurrence patterns of TSG responsible behaviors among short video app users while also enhancing the recognition of the interaction between different antecedent variables. Thus, Study 2 contributes to the accurate identification of the contextual environment of variable influence effects and functional logic, providing valuable extensions to the integrated VBN and TPB model.

### 4.2. Managerial Implications

This study offers several practical implications. First, users who have watched TSG-related short videos often possess a more positive CW, higher awareness of consequence, and a stronger sense of responsibility. These values, beliefs, and norms increase their likelihood of engaging in TSG responsible behaviors. By analyzing user similarities and leveraging advanced algorithmic technologies to match shared interests, short video platforms are able to use collaborative filtering algorithms based on user profiles to push personalized content. Additionally, short video platforms skillfully integrate user preferences with big data from mobile social networks, relying on efficient algorithmic recommendation systems to achieve deep customization and wide dissemination of content. This mechanism, based on users’ interactive feedback and viewing behavior data, automatically elevates highly attractive video content to higher-level distribution channels, effectively promoting viral information dissemination and broad exposure to high-quality content. Thus, despite the differences in algorithm implementation across short video platforms in different regions, they all demonstrate significant effectiveness in enhancing TSG exposure and public attention. Therefore, countries should actively explore innovative approaches, fully leveraging mainstream short video platforms, such as TikTok, YouTube Shorts, and Instagram, to deeply integrate local TSG elements with short video media, thus creating new models for protection, display, transmission, and development (Ramon & Rojas-Torrijos, 2022).

Furthermore, TSGs worldwide, such as cricket in the UK or flamenco dance in Spain, can fully utilize short video platforms for dissemination and protection. Specific strategies include systematically incorporating heritage protection responsibility concepts into short video content, enhancing creative design of relevant elements, and particularly emphasizing the sense of responsibility and ethical principles associated with TSG protection and transmission. Such an approach guides users in forming positive cultural attitudes and behavioral patterns. Additionally, deeply exploring the cultural connotations and values of TSGs is essential, using scenario simulations and problem warnings to raise public awareness of the urgency of TSG protection. Doing so can inspire the public’s intrinsic motivation to take positive protective measures. Furthermore, TSG inheritors should be encouraged to use short videos to document their daily training, competition moments, and the stories behind them, showcasing their unique skills and cultural heritage to larger audiences. By initiating challenges and topic activities centered around TSG transmission, users are encouraged to imitate, innovate, and share their own TSG experiences, fostering widespread user participation and topic discussion. By collaborating with educational institutions, cultural organizations, and tourism departments to launch combined online and offline experiences, such as virtual tours and workshops, TSG dissemination channels and audience bases can be further broadened, ultimately achieving the goal of cultivating national TSG responsible behaviors.

However, practical challenges must be addressed. First, the transparency of platform algorithms is a critical issue. Many short video platforms operate their recommendation algorithms as “black boxes”, making it difficult for users to understand how content is actually curated, specifically how TSG-related content is promoted. This lack of transparency could result in insufficient exposure for TSG content, which may be replaced by more entertainment-oriented content, thereby hindering the accurate transmission of cultural heritage.

Second, internal priority conflicts within platforms may arise. To pursue commercial interests, platforms tend to prioritize content that is popular and generates more engagement, while TSG content is often overlooked. This conflict between commercial interests and cultural protection limits the widespread dissemination of TSGs and requires a balanced approach to ensure that both types of content can coexist and thrive.

Finally, evolving regulatory environments also present challenges. Differences in cultural heritage protection laws across various countries and regions impose compliance pressures on short video platforms when promoting TSG globally. Furthermore, increasingly stringent data privacy regulations may limit platforms’ ability to use personal data for algorithmic personalization, thus affecting the effectiveness of content recommendation systems. In such a dynamic regulatory environment, platforms must be adaptable, adjusting their strategies to ensure the continuous dissemination of TSG content.

Notably, perceived behavioral control, moral norm, and subjective norm are also key factors that directly influence TSG responsible behaviors among short video app users. This insight opens up new avenues for promoting TSG responsible behaviors. From the perspective of perceived behavioral control, short video platforms should strive to create a low-threshold but high-reward creative and dissemination environment, thus reducing the difficulty and cost of user participation in TSG content creation through technological optimization and resource allocation. For example, developing specialized TSG content creation tools, providing templates, material libraries, and editing guidance ensure that users can easily get started. All of these effectively increase the quantity and quality of content, thereby enhancing users’ perceptions of their ability to positively influence TSG dissemination. On the level of moral norm, platforms can deeply explore the cultural value and ethical significance behind TSGs, thus leveraging the influence of social media by inviting celebrities, cultural scholars, and TSG inheritors to serve as “opinion leaders”, making positive statements to strengthen societal moral consensus on TSG protection. Finally, regarding subjective norms, platforms should encourage positive interactions and feedback mechanisms among users. These efforts may include setting up functions for likes, comments, and shares, enabling users to feel supported and recognized by the community and thus enhancing their subjective willingness to adhere to moral norm and participate in TSG protection. Nevertheless, challenges remain in ensuring that users’ engagement is not only genuine but sustained. Overcoming issues, such as user apathy and platform fatigue, requires continuous innovation in how TSG content is presented and incentivized.

Finally, the occurrence of TSG responsible behaviors is closely related to the variables in the theoretical model. However, the influence of these antecedent variables on TSG responsible behaviors must be realized through mediating mechanisms. Specifically, CW, awareness of consequence, and ascription of responsibility influence TSG responsible behaviors through mediating variables, including perceived behavioral control, attitude toward behavior, subjective norm, and moral norm. Therefore, to transform CW, awareness of consequence, and ascription of responsibility into actual TSG responsible behaviors, short video platforms and users should focus on the intermediary conditions for the transition of these three variables to TSG responsible behaviors. Through publicity, education, and information push mechanisms, these mediating factors should be effectively regulated to maximize the role of CW, awareness of consequence, and ascription of responsibility in the formation of heritage responsible behaviors. For example, users can be subtly influenced to develop a positive attitude toward behavior and enhance their moral norm recognition by using the push mechanism of short video platforms, regularly conveying the value significance of TSG protection to users, and emphasizing its positive impact on social development. In TSG short video creation, the cultural heritage and value connotations of TSG projects should be deeply explored, and users’ subjective norm levels should be enhanced through interaction. By sharing success stories and providing positive feedback, short video app users’ self-efficacy in engaging in TSG responsible behaviors can be strengthened, boosting their confidence in engaging in TSG responsible behaviors, enhancing their perceived behavioral control levels, and strengthening their TSG responsible behaviors. However, to ensure the sustainability of these efforts, careful consideration of regulatory and platform-specific challenges is needed. This is because regulatory environments may affect the ability of platforms to carry out these initiatives, and internal conflicts between platform goals (commercial versus cultural preservation) might hinder the implementation of certain strategies.

### 4.3. Research Limitations and Future Prospects

First, as a topic of global concern, the protection of cultural heritage embodies fundamental values that hold a certain degree of universality. Although the theoretical model proposed in this study has been supported within the context of China, cultural differences in value orientations and social norms may influence the motivational structure of responsible behaviors. Thus, to enhance the credibility and cross-cultural applicability of the findings, future research could incorporate cross-cultural samples and collect relevant objective data to further validate the conclusions.

Second, the diversity of TSG content on short video apps is extremely high. Given that the type and amount of content viewed by users may have a significant impact on the research outcomes, future research should fully consider the nature and quality of short video content. In particular, such studies must assess the impact of short video apps on users’ sense of responsibility from a comprehensive perspective. Furthermore, the cultural background of TSGs and events might influence users’ sense of responsibility differently depending on the region. Future studies should consider the variations in cultural backgrounds to enhance the universality and applicability of the research conclusions.

## Figures and Tables

**Table 1:** Descriptive statistics, reliability, and convergent validity.

Variable	Item	Mean (Std. Dev.)	Skewness	Kurtosis	Factor Loadings	Cronbach’s Alpha	CR	AVE
Cultural linkage	CL1	5.2 ± 1.32	−0.434	−0.274	0.900	0.854	0.911	0.773
	CL2	5.02 ± 1.365	−0.566	0.165	0.864			
	CL3	4.73 ± 1.548	−0.381	−0.486	0.874			
Recognition of cultural value	RC1	5.44 ± 1.233	−0.777	0.782	0.860	0.873	0.913	0.724
	RC2	5.15 ± 1.216	−0.598	0.394	0.845			
	RC3	5.28 ± 1.197	−0.643	0.551	0.812			
	RC4	5.26 ± 1.175	−0.631	0.549	0.886			
Loss sensitivity	LS1	4.98 ± 1.65	−0.543	−0.559	0.893	0.846	0.907	0.765
	LS2	4.84 ± 1.432	−0.568	−0.093	0.877			
	LS3	5.06 ± 1.378	−0.676	0.358	0.853			
Preservation consistency	PC1	5.46 ± 1.365	−1.01	0.898	0.901	0.859	0.914	0.781
	PC2	5.24 ± 1.448	−0.926	0.518	0.888			
	PC3	5.34 ± 1.373	−0.96	0.772	0.860			
Awareness of consequence	AC1	5.29 ± 1.358	−0.609	0.003	0.890	0.826	0.896	0.742
	AC2	5.2 ± 1.3	−0.596	0.186	0.847			
	AC3	5.1 ± 1.37	−0.621	0.095	0.847			
Perceived behavioral control	PBC1	5.5 ± 1.231	−0.708	0.236	0.889	0.908	0.936	0.784
	PBC2	5.18 ± 1.231	−0.565	0.069	0.879			
	PBC3	5.36 ± 1.195	−0.649	0.254	0.880			
	PBC4	5.15 ± 1.29	−0.464	−0.113	0.895			
Attitude toward behavior	ATB1	5.26 ± 1.527	−0.805	0.132	0.905	0.886	0.929	0.814
	ATB2	5.4 ± 1.297	−1.101	1.738	0.906			
	ATB3	5.36 ± 1.343	−0.743	0.275	0.896			
Subjective norm	SN1	5.03 ± 1.47	−0.496	−0.319	0.921	0.898	0.936	0.831
	SN2	5.01 ± 1.415	−0.456	−0.303	0.920			
	SN3	5.16 ± 1.255	−0.37	−0.292	0.893			
Ascription of responsibility	AR1	5.22 ± 1.407	−0.927	0.687	0.874	0.837	0.902	0.754
	AR2	5.29 ± 1.401	−0.901	0.518	0.854			
	AR3	5.22 ± 1.421	−0.869	0.376	0.877			
Moral norm	MN1	5.47 ± 1.259	−0.812	0.682	0.867	0.881	0.918	0.737
	MN2	5.16 ± 1.189	−0.65	0.484	0.865			
	MN3	5.31 ± 1.172	−0.68	0.638	0.819			
	MN4	5.31 ± 1.17	−0.608	0.488	0.882			
TSG responsible behavior	TRB1	5.04 ± 1.555	−0.557	−0.429	0.899	0.911	0.937	0.789
	TRB2	4.84 ± 1.44	−0.479	−0.345	0.876			
	TRB3	4.99 ± 1.374	−0.605	0.187	0.878			
	TRB4	4.87 ± 1.461	−0.49	−0.22	0.899			

Note: ATB = attitude toward behavior; PBC = perceived behavioral control; SN = subjective norm; AR = responsibility attribution; MN = moral norm; AC = awareness of consequence; TRB = TSG responsible behavior; CW = cultural worldview; CL = cultural affiliation; RC = cultural recognition; LS = loss sensitivity; PC = preservation consistency, the following are the same.

**Table 2:** Discriminant validity.

	AC	AR	ATB	CL	TRB	LS	MN	PBC	PC	RC	SN
AC	0.861										
AR	0.676	0.868									
ATB	0.635	0.460	0.902								
CL	0.482	0.351	0.321	0.879							
TRB	0.500	0.527	0.541	0.423	0.888						
LS	0.574	0.433	0.417	0.452	0.448	0.874					
MN	0.464	0.634	0.323	0.367	0.620	0.395	0.859				
PBC	0.643	0.485	0.493	0.360	0.542	0.421	0.378	0.886			
PC	0.585	0.494	0.449	0.527	0.426	0.497	0.445	0.518	0.883		
RC	0.463	0.360	0.376	0.459	0.360	0.416	0.341	0.368	0.515	0.851	
SN	0.434	0.520	0.362	0.542	0.662	0.425	0.653	0.365	0.444	0.434	0.911

**Table 3:** Structural model analysis results.

Variable	*R* ^2^	*Q* ^2^	GOF
Awareness of consequence	0.473	0.343	0.612
Ascription of responsibility	0.457	0.342	
Attitude toward behavior	0.404	0.324	
Moral norm	0.545	0.398	
Perceived behavioral control	0.414	0.321	
TSG responsible behavior	0.617	0.482	

**Table 4:** Path coefficients.

Path	Path Coefficient (*β*)	STDEV	T	*p*	Hypothesis
CL → AC	0.125	0.049	2.575	0.010	Confirmed
LS → AC	0.319	0.044	7.341	0.000	Confirmed
PC → AC	0.298	0.054	5.496	0.000	Confirmed
RC → AC	0.119	0.051	2.336	0.020	Confirmed
AC → AR	0.676	0.037	18.228	0.000	Confirmed
AC → ATB	0.635	0.036	17.701	0.000	Confirmed
AC → PBC	0.643	0.037	17.348	0.000	Confirmed
AR → MN	0.403	0.044	9.095	0.000	Confirmed
SN → MN	0.444	0.047	9.482	0.000	Confirmed
MN → TRB	0.241	0.038	6.368	0.000	Confirmed
PBC → TRB	0.210	0.036	5.841	0.000	Confirmed
ATB → TRB	0.235	0.036	6.459	0.000	Confirmed
SN → TRB	0.342	0.038	8.998	0.000	Confirmed

**Table 5:** Impact effects of antecedent variables.

Antecedent Variables	The Impact of TSG Responsible Behavior		
	Direct Effect	Indirect Effect	Total Effect
CL	-	0.044 **	0.044 **
LS	-	0.112 ***	0.112 ***
PC	-	0.104 ***	0.104 ***
RC	-	0.042 **	0.042 **
AC	-	0.350 ***	0.350 ***
SN	0.342 ***	0.107 ***	0.449 ***
AR	-	0.097 ***	0.097 ***
ATB	0.235 ***	-	0.235 ***
MN	0.241 ***	-	0.241 ***
PBC	0.210 ***	-	0.210 ***

Note: “-” indicates no effect; * indicates *p* < 0.1, ** indicates *p* < 0.05, and *** indicates *p* < 0.01.

**Table 6:** Necessity analysis of individual antecedent conditions.

Antecedent Condition	Outcome Condition: TRB		Antecedent Condition	Outcome Condition: TRB	
	Consistency	Coverage		Consistency	Coverage
CW	0.920487	0.853514	~CW	0.347659	0.867391
AC	0.909831	0.848483	~AC	0.344058	0.845402
AR	0.920644	0.845421	~AR	0.320559	0.821312
PBC	0.927632	0.843668	~PBC	0.320429	0.843775
ATB	0.930541	0.841105	~ATB	0.305203	0.818352
SN	0.910122	0.883884	~SN	0.363927	0.809457
MN	0.941510	0.848368	~MN	0.314466	0.851081

Note: ~ represents logical “NOT”, indicating the opposite condition. For instance, “~Cultural Worldview” denotes the absence or low level of cultural worldview.

**Table 7:** Sufficiency analysis of antecedent condition combinations.

Predictive Configurations of TSG Responsible Behavior	Raw Coverage	Unique Coverage	Consistency
SN*MN*ATB*PBC*CW	0.7925	0.0212	0.9535
SN*MN*ATB*AR*CW	0.7933	0.0219	0.9519
SN*MN*PBC*AR*CW	0.7882	0.0169	0.9465
SN*MN*ATB*PBC*AR*AC	0.7715	0.0093	0.9556
SN*ATB*PBC*AR*AC*CW	0.7744	0.0122	0.9584
MN*ATB*PBC*AR*AC*CW	0.7915	0.0293	0.9460
Overall Coverage: 0.8822			
Overall Consistency: 0.9200			

Note: “*” represents logical “AND”; the same applies below.

**Table 8:** Sufficiency analysis of antecedent condition combinations after adjusting calibration thresholds.

Predictive Configurations of TSG Responsible Behavior	Raw Coverage	Unique Coverage	Consistency
SN*MN*ATB*PBC*CW	0.7708	0.0248	0.9419
SN*MN*ATB*AR*CW	0.7735	0.0275	0.9389
SN*MN*PBC*AR*CW	0.7631	0.0171	0.9339
SN*MN*ATB*PBC*AR*AC	0.7445	0.0099	0.9436
SN*ATB*PBC*AR*AC*CW	0.7486	0.0139	0.9474
MN*ATB*PBC*AR*AC*CW	0.7720	0.0374	0.9307
Overall Coverage: 0.8766			
Overall Consistency: 0.9029			

Note: “*” represents logical “AND”; the same applies below.

## Data Availability

The raw data supporting the conclusions of this manuscript will be made available by the authors to any qualified researcher.

## Abbreviations

The following abbreviations are used in this manuscript:

TSGs	Traditional sports and games
VBN	Value-belief-norm theory
TPB	Theory of planned behavior
PLS-SEM	Partial least squares structural equation modeling
FsQCA	Fuzzy-set qualitative comparative analysis
AVE	Average variance extracted

## Appendix A

**Table A1:** Variables and measurement items.

Latent Variable	Measurement Items	References
Cultural Linkage (CL)	CL1: The TSGs passed down by my ancestors are important to me.	(Choi et al., 2009)
	CL2: TSGs enhance my sense of individual identity and pride.	
	CL3: The TSGs we enjoy should be preserved for future generations.	
Recognition of Cultural Value (RC)	RC1: TSGs do not enhance my sense of individual identity. (-)	(Choi et al., 2009)
	RC2: TSGs do not make me happier. (-)	
	RC3: Students do not need to learn about TSGs. (-)	
	RC4: We do not need to care about TSGs. (-)	
Loss Sensitivity (LS)	LS1: We are not currently losing our TSGs. (-)	(Choi et al., 2009)
	LS2: There is a trend of TSGs disappearing.	
	LS3: If the current situation does not improve, we will soon lose most of our TSGs.	
Preservation Consistency (PC)	PC1: I want to learn more about the TSGs passed down by my ancestors.	(Choi et al., 2009)
	PC2: We need to preserve the beliefs and customs from past TSGs.	
	PC3: TSGs hold no significance to me. (-)	
Awareness of Consequence (AC)	AC1: The dissemination of TSGs through short videos will positively impact its preservation.	(Kiatkawsin & Han, 2017)
	AC2: Users who watch TSG short videos will influence those around them.	
	AC3: Short videos help maximize the protection and dissemination of TSGs.	
Perceived Behavioral Control (PBC)	PBC1: Whether to take action to protect the TSGs depicted in the videos depends on me.	(Ajzen, 1991); (H. Han, 2015)
	PBC2: If I am willing, I can protect the TSGs depicted in the videos.	
	PBC3: I have the financial resources, time, or opportunities to protect the TSGs depicted in the videos.	
	PBC4: It is easy for me to protect the TSGs depicted in the videos.	
Attitude Toward Behavior (ATB)	ATB1: Protecting the TSGs depicted in the videos is a positive behavior.	(Ajzen, 1991)
	ATB2: Protecting the TSGs depicted in the videos is a necessary behavior.	
	ATB3: Protecting the TSGs depicted in the videos is a meaningful behavior.	
Subjective Norm (SN)	SN1: Important people in my life think I should protect the TSGs depicted in the videos.	(Ajzen, 1991); (H. Han, 2015)
	SN2: Important people in my life want me to protect the TSGs depicted in the videos.	
	SN3: People whose opinions I value expect me to protect the TSGs depicted in the videos.	
Ascription of Responsibility (AR)	AR1: People are responsible for the impact their daily activities have on TSGs.	(H. Han, 2015; Kiatkawsin & Han, 2017)
	AR2: People are responsible for the damage their daily activities cause to TSGs.	
	AR3: People must take responsibility for the problems they cause to TSGs.	
Moral Norm (MN)	MN1: I have a duty to protect the TSGs depicted in the videos.	(Onwezen et al., 2013)
	MN2: I should protect the TSGs depicted in the videos.	
	MN3: It is generally believed that protecting the TSGs depicted in the videos is important.	
	MN4: I have a duty to engage in behaviors that benefit the TSGs depicted in the videos.	
TSG Responsible Behavior (TRB)	TRB1: I will learn more about the TSGs depicted in the videos.	(Gursoy et al., 2019)
	TRB2: I will discuss the TSGs depicted in the videos with my friends and family.	
	TRB3: I will participate in activities to protect the TSGs depicted in the videos.	
	TRB4: I feel responsible for protecting the TSGs depicted in the videos.	

Note: (-) indicates reverse-scored items.
